# Reduction in Retinal Ganglion Cell Layer, Inner Plexiform Layer, and Choroidal Thickness in Children With Autism Spectrum Disorder

**DOI:** 10.7759/cureus.49981

**Published:** 2023-12-05

**Authors:** Mahmut Zabit Kara, Mehmet Hamdi Örüm, Ayşe Sevgi Karadağ, Aysun Kalenderoğlu, Aslıhan Kara

**Affiliations:** 1 Child Adolescent Psychiatry, University of Health Sciences, Antalya, TUR; 2 Psychiatry, Elazığ Mental Health and Diseases Hospital, Elazığ, TUR; 3 Ophthalmology, Dünyagöz Istanbul, Istanbul, TUR; 4 Psychiatry, Private Clinic, Izmir, TUR; 5 Biological Sciences, Semikal Technology, Antalya, TUR

**Keywords:** optical coherence tomography (oct), inner plexiform layer, autism spectrum disorder, choroidal layer, ganglion cell layer

## Abstract

Purposes: The aim of this study was to evaluate the retinal nerve fiber layer (RNFL), choroidal layer, inner plexiform layer (IPL), and ganglion cell layer (GCL) in patients with autism spectrum disorder (ASD).

Methods: In this study, we measured the thickness of the RNFL, GCL, IPL, and choroidal thickness using a spectral optical coherence tomography (OCT) device and we compared the results between the children diagnosed with ASD and healthy controls. Correlation between the Childhood Autism Rating Scale (CARS) and the OCT data was evaluated.

Results: Both ASD and control group consisted of 40 subjects (30 males and 10 females). Of the children in the ASD group, 29 had normal intelligence and 11 had mild intellectual disability (MID). The mean age of patients in the ASD group and control groups were 9.77 ± 3.37 years and 9.85 ± 3.97 years (p = 0.928). There was a statistically significant difference between the ASD group and the control group in the nasal and nasal-superior sectors of the RNFL layers in the left eye when all the lower layers of RNFL were assessed. In both eyes, the children with ASD had considerably lower mean choroidal thicknesses than the controls. When compared to the controls, the GCL and IPL volumes in the individuals with ASD were considerably lower in both eyes. Compared to the MID group, the left GCL volume of the nasal-inferior group was noticeably higher. A significant correlation was found between CARS scores and left GCL left IPL.

Conclusions: In contrast to RNFL in the ASD group, significant reductions in IPL, GCL, and choroidal thickness were observed in both eyes. It is thought that GCL may be a much more important biomarker than RNFL in terms of representing the structural deterioration in the brain. In addition, these results may form the basis for a new perspective on the use of OCT for the diagnosis and clinical course of autism.

## Introduction

Autism spectrum disorder (ASD) is a neurodevelopmental disorder that first appears in early childhood and is characterized by repetitive behaviors, limited interests, and persistent impairment in social and communication skills [1]. According to the Centers for Disease Control and Prevention, the prevalence of ASD was reported to be 1/150, 1/59, and 1/36 in 2000, 2014, and 2020, respectively, in the United States, and a striking increase in the incidence of ASD has been revealed [2].

Numerous neuroimaging techniques have been used to dissect the neurophysiological mechanisms underlying ASD and offer crucial insights into the alterations in anatomy, function, and neurochemistry. Functional magnetic resonance imaging (MRI) has been used to show damaged functional brain networks, while longitudinal structural MRI has identified an aberrant developmental trajectory of ASD that is linked to cascade neurobiological processes [3]. There are also studies that found abnormalities in grey matter [4] and white matter [5] as well as deterioration in neural connections. Conflicting results have been reported in studies investigating cortical thickness in ASD, reporting both increases and decreases in a variety of measures. The divergent outcomes seem to some extent to be due to the fact that ASD is a very heterogeneous disorder, characterized by a wide spectrum of symptoms of varying severity and often accompanied by one of a number of comorbid conditions. In addition to elucidating the neurophysiological basis of ASD, studies are needed to find diagnostic and prognostic biomarkers [6].

A high-resolution, non-invasive imaging method called optical coherence tomography (OCT) is able to produce cross-sectional images of retinal tissue. The retina and optic nerve are components of the central nervous system because they grow from the diencephalon during embryonic development. Thus, the retina and optic nerve can be thought of as a “window to the brain” because they share characteristics and relationships with the brain in terms of embryology, architecture, physiology, and histology. Similar to how the brain is made up, the retina has both white matter and grey matter. As a result, it is believed that changes in the structure or function of the brain could have an impact on the retina [7]. It has been reported that the ganglion cell layer (GCL), the inner plexiform layer (IPL), and the retinal nerve fiber layer (RNFL) are related to the overall volume of the brain's grey and white matter [8]. In recent years, OCT has been widely studied in neurodegenerative diseases such as multiple sclerosis [9], Alzheimer's [10], and Parkinson's disease [11], and RNFL thinning has been associated with neurodegeneration, disease severity, and low functionality [12]. In addition, changes in GCL, IPL, RNFL, and choroidal thickness have been investigated in psychiatric disorders [7,12].

Studies investigating OCT parameters in ASD are limited. In addition, different results related to RNFL have been reported in studies. While Emberti-Gialloreti et al. [13] showed a decrease in nasal quadrant RNFL thickness in a study with 24 adult patients, Garcia-Medina et al. [14] found an increased thickness in all layers in children and adolescents. Contrary to these two studies, Little et al. [15] found no relationship between RNFL thickness and ASD. Friedel et al. [16] found no difference in adult patients in terms of RNFL but showed reduced overall macula and outer nuclear layer thickness and volume. These changes were found to be associated with autism symptom severity. Finally, the RNFL sub-sectors were all considerably lower in the ASD group compared to the control group, according to Bozkurt et al. [17], although there was no association between RNFL thickness and the severity of the ASD symptoms.

The aim of this study was to obtain detailed information about possible structural retinal changes in children with ASD. Due to the inconclusive results of previous research, we wanted to clarify whether the RNFL was thicker, thinner, or unaltered. We also aimed to compare choroidal thickness between children with ASD and healthy controls, as well as other intra-retinal layers (GCL, IPL). To the best of our knowledge, this is the first study to investigate all of these parameters in ASD. In addition, we aimed to determine whether there is a relationship between retinal changes and autism symptom severity.

## Materials and methods

Our study was a single-center, retrospective, implemented using a controlled design. Detailed sociodemographic and clinical data of all patients in the pediatric age group followed in our clinic are recorded in the patient record system. Among the ASD patients followed in the Child and Adolescent Psychiatry Outpatient Clinic of the Faculty of Medicine, those who underwent OCT evaluation during routine eye examinations were scanned and 40 patients who met the criteria were included in the study. Patients who had comorbid first axis diagnosis, hypertension, diabetes mellitus, severe neurological, immunological, or systemic diseases (glaucoma or retinal diseases), and drug use were excluded. Patients with refraction errors ≥ 1 prism diopter were also excluded. Both the patient and the control groups were examined in the ophthalmology clinic and best-corrected visual acuity, intraocular pressure, slit-lamp bio-microscopy, and fundus examination by eye dilatation were measured. Patients and controls with normal eye findings were included. The group of healthy controls did not have any first axis diagnosis, hypertension, diabetes mellitus, severe neurological, immunological, or systemic diseases, or drug use which may affect the results. The Childhood Autism Rating Scale (CARS) was routinely applied to children diagnosed with ASD at every clinic admission, and these data were used in the study [18]. CARS is a widely used rating scale for the detection and diagnosis of ASD [18]. The CARS consists of 14 domains assessing behaviors associated with autism, with a 15th domain rating general impression of autism. Each domain is scored on a scale ranging from one to four; higher scores are associated with a higher level of impairment. The scores of the scale allow us to determine the severity of autism as mild to moderate and severe autism. The Schedule for Affective Disorders and Schizophrenia for School-Age Children-Present Version (KSADS-P) and the Wechsler Intelligence Scale for Children-Revised (WISC-R) data applied to the patients were also accessed through the patient record system [19]. A total of 160 eyes, 80 in the ASD group and 80 in the control group were included in the evaluation. Local ethics committee approval was obtained.

A spectral-OCT device (SpectralisTM OCT, Version 6.0, Heidelberg Engineering, Germany) was used to assess the RNFL and choroid thicknesses and GCL and IPL volumes in both eyes. The RNFL includes temporal (T), nasal (N), temporal superior (TS), temporal inferior (TI), and global (mean) segments. Therefore, seven measurements were made for each eye (i.e., N, NS, NI, T, TS, TI, mean). The choroid structure was also measured with OCT. The choroidal thickness was measured manually. A perpendicular line was drawn subfoveal from the outer edge of the retinal pigment epithelium to the choroid-sclera junction. Two additional lines were drawn at the nasal and temporal sides at 500 µm intervals from the subfoveal line. The mean value of these three measures was accepted as the choroidal thickness. All measurements were performed by the same author who was blinded to the diagnoses of the patients. The choroidal measurement method used with the spectral-OCT devices has been previously explained. Lastly, we measured the GCL and IPL volumes with an OCT device.

Statistical analyses were performed using the SPSS 22.0 package program (IBM Corp., Armonk, NY). The mean ± standard deviation and percentages were used as descriptive statistics. The Chi2 test was used to compare categorical variables. The normality of the data was tested using the Kolmogorov-Smirnov test. An independent samples t-test was used to compare two normally distributed variables and the Mann-Whitney U test was used to compare two non-normally distributed variables. Pearson correlation analysis was performed in the ASD group. A value of less than 0.05 (p-value) was considered statistically significant.

## Results

Both ASD and control groups consisted of 40 subjects (30 males and 10 females). The mean patient ages in the ASD group and control groups were 9.77 ± 3.37 and 9.85 ± 3.97 years, respectively, and it was not significant (p = 0.928). Of the patients in the ASD group, 29 had normal intelligence (NI) and 11 had mild intellectual disability (MID). Comparison of ASD and control groups in terms of RNFL values were shown in Table 1.

**Table 1:** Comparison of ASD and control group in terms of RNFL values *p<0.05 Note: Unit is μm Abbreviations: ASD: Autism Spectrum Disorder; RNFL: Retinal Nerve Fiber Layer; SD: Standard Deviation; NS: Naso-Superior; NI: Naso-Inferior; N: Nasal; TS: Temporo-Superior; TI: Temporo-Inferior; T: Temporal

RNFL Parameters	ASD (n=40) Mean ± SD		Control (n=40) Mean ± SD		P-values	
	Right	Left	Right	Left	Right	Left
NS	119.17 ± 15.53	121.77 ± 15.14	130.47 ± 19.09	151.50 ± 19.61	0.950	0.023*
NI	119.10 ± 23.71	121.20 ± 23.29	117.32 ± 25.31	119.22 ± 28.07	0.791	0.733
N	80.30 ± 21.40	70.67 ± 12.56	78.37 ± 23.51	89.05 ± 14.53	0.703	0.016*
TS	146.55 ± 16.99	145.07 ± 20.56	146.12 ± 19.08	136.72 ± 18.59	0.917	0.060
TI	148.40 ± 23.76	139.97 ± 32.71	148.90 ± 22.82	138.85 ± 33.15	0.924	0.879
T	79.51 ± 15.33	74.50 ± 12.10	74.85 ± 8.58	71.85 ± 11.90	0.100	0.327
Mean	108.35 ± 17.58	102.27 ± 9.28	106.35 ± 11.00	108.57 ± 9.49	0.544	0.080

In the ASD group, the choroidal layer, IPL, and GCL thickness were significantly lower in both eyes (p<0.05). Table 2 displays the choroidal thickness, GCL, and IPL volumes of both the Autism ASD group and the control group.

**Table 2:** Choroidal thickness, GCL, and IPL volumes of ASD and control groups *p<0.05 Note: Unit is μm Abbreviations: ASD: Autism Spectrum Disorder; GCL: Ganglion Cell Layer, IPL: Inner Plexiform Layer, SD: Standard Deviation

	ASD (n=40) Mean ± SD	Control (n=40) Mean ± SD	P-values
Right Choroid	316.24 ± 44.62	352.26 ± 45.35	0.014*
Left Choroid	316.35 ± 35.87	346.19 ± 32.12	0.017*
Right GCL	1.08 ± 0.08	1.19 ± 0.08	0.000*
Left GCL	1.09 ± 0.09	1.17 ± 0.08	0.000*
Right IPL	0.88 ± 0.06	0.96 ± 0.06	0.000*
Left IPL	0.90 ± 0.06	0.95 ± 0.06	0.000*

The ASD group was divided into two categories: those with normal intelligence (n: 29) and those with mild intellectual disability (n: 11). These two groups were compared in terms of OCT parameters. The RNFL values of these two groups are presented in Table 3, while the values for choroidal thickness, IPL, and GCL are presented in Table 4. The only distinction in terms of RNFL was a significant thinning in the temporal sector of the right eye in the ASD group with intellectual disability (p: 0.030). Apart from this, no differences were detected in terms of RNFL. 

**Table 3:** Comparison of the ASD group with Normal Intelligence (NI) and with Mild Intellectual Disability (MID) in terms of RNFL layers *p<0.05 Note: Unit is μm Abbreviations: ASD: Autism Spectrum Disorder; RNFL: Retinal Nerve Fiber Layer; SD: Standard Deviation; NS: Naso-Superior; NI: Naso-Inferior; N: Nasal; TS: Temporo-Superior; TI: Temporo-Inferior; T: Temporal

RNFL Parameters	NI (n=29) Mean ± SD		MID (n=11) Mean ± SD		P-values	
	Right	Left	Right	Left	Right	Left
NS	119.51 ±17.17	122.20 ± 16.12	118.27 ±10.70	120.63 ± 11.02	0.786	0.732
NI	119.48 ±18.96	118.34 ± 17.99	118,09 ±13.38	128.72 ± 14.11	0.867	0.212
N	82.06 ±14.32	71.06 ± 14.98	75.63 ±9.88	69.63 ± 9.97	0.242	0.714
TS	149.06 ±17.26	146.06 ± 17.32	139.90 ±14.99	142.45 ± 14.99	0.110	0.626
TI	148.96 ±22.04	136.41 ± 22.32	146.90 ±21.02	149.36 ± 20.99	0.796	0.269
T	82.69 ±15.79	75.65 ± 15.46	72.00 ±11.57	71.45 ± 11.67	0.030*	0.318
Mean	110.62 ±9.81	102.00 ± 9.97	102.36 ±7.22	103.00 ± 7.22	0.061	0.765

In terms of choroidal thickness, IPL, and GCL values, the thickness of GCL was significantly lower in the left eye in the group with intellectual disability (p: 0.012). Other parameters were similar in both groups.

**Table 4:** Choroidal thickness, GCL, and IPL volumes of ASD group with normal intelligence (NI) and with mild intellectual disability (MID) *p<0.05 Note: Unit is μm Abbreviations: ASD: Autism Spectrum Disorder; GCL: Ganglion Cell Layer, IPL: Inner Plexiform Layer, SD: Standard Deviation

	NI (n=29) Mean ± SD	MID (n=11) Mean ± SD	P-values
Right Choroid	320.92 ± 43.42	304.33 ± 46.02	0.354
Left Choroid	314.43 ± 35.83	321.23 ± 33.01	0.683
Right GCL	1.09 ± 0.09	1.07 ± 0.08	0.551
Left GCL	1.11 ± 0.08	1.04 ± 0.08	0.012*
Right IPL	0.87 ± 0.06	0.88 ± 0.06	0.735
Left IPL	0.91 ± 0.06	0.87 ± 0.06	0.084

The relationship between ASD symptom severity and OCT parameters is presented in Table 5. While a significant negative correlation was found between the thickness of the left IPL (p: 0.036, r: -0.337), left GCL (p: 0.013, r: -0.396), and disease severity, no differences were observed in terms of other parameters.

**Table 5:** Correlation between ASD symptom severity and OCT parameters *p<0.05 Abbreviations: ASD: Autism Spectrum Disorder; CARS: Childhood Autism Rating Scale; NS: Naso-Superior; NI: Naso-Inferior; N: Nasal; TS: Temporo-Superior; TI: Temporo-Inferior; T: Temporal; GCL: Ganglion Cell Layer; IPL: Inner Plexiform Layer; r: Pearson correlation coefficient; p: significance value

		CARS
Right NS	r	-0.670
	p	0.681
Right NI	r	0.131
	p	0.422
Right N	r	-0.037
	p	0.820
Right TS	r	-0.246
	p	0.126
Right TI	r	0.057
	p	0.727
Right T	r	-0.234
	p	0.164
Right Mean	r	-0.054
	p	0.742
Left NS	r	-0.197
	p	0.224
Left NI	r	0.279
	p	0.081
Left N	r	-0.051
	p	0.754
Left TS	r	-0.087
	p	0.595
Left TI	r	0.005
	p	0.978
Left T	r	-0.149
	p	0.359
Left Mean	r	-0.032
	p	0.845
Right Choroid	r	-0.049
	p	0.767
Left Choroid	r	0.014
	p	0.933
Right GCL	r	-0.203
	p	0.215
Left GCL	r	-0.396
	p	0.013*
Right IPL	r	-0.113
	p	0.493
Left IPL	r	-0.337
	p	0.036*

## Discussion

In our study, subjects with ASD as well as healthy controls were examined in terms of RNFL, GCL, IPL, and choroidal thickness in both eyes, and their relationship with intelligence and disease severity was examined. In the ASD group, the RNFL thickness was significantly reduced in the left nasal and left nasal superior quadrants, while the global RNFL thickness was similar between the groups. Secondly, GCL, IPL, and choroid thickness were significantly reduced in both eyes in the ASD group. Lastly, in cases with ID accompanying ASD, the GCL was significantly thinner in the left eye compared to those with normal intelligence, and a more severe thinning in the left GCL and left IPL was associated with increased symptom severity.

Because of their shared biological ancestry and neurodevelopmental development from the ectoderm, the retina, and brain tissue are both central nervous system components that open outward. As a result, it is believed that any changes in the structure or operations of the brain may have an impact on the retina. Given that the axons of ganglion cells within the retina are not myelinated, RNFL thinning is hypothesized to serve as a proxy marker of neuronal loss [20]. Indeed, defects in neuronal development or chronic axonal degeneration processes are frequently associated with RNFL thinning [21].

Since ASD is a neurodevelopmental disorder characterized by defects in neurogenesis, neural migration, and other defects in neuronal structures, studies have been conducted with the hypothesis that changes in the RNFL may represent some aspect of these abnormalities. Conflicting results have been reported in studies examining RNFL in patients with ASD. Emberti-Giollerati et al. [13] found a significant reduction in total RNFL thickness in young adult patients with High-Functioning Autism, whereas Garcia-Medina et al. [14] found a thickening in the global RNFL in children. Little et al. [15], on the other hand, stated that there was no significant difference between the ASD and control groups in terms of RNFL. Finally, Bozkurt et al. [17] found thinning in total RNFL with temporal, temporal superior, temporal inferior, and nasal superior quadrants in children. The differences between the results were related to factors such as the choice of the patient group, the mean age of the patients, and the resolution of the OCT device used. In our study, the mean age of the patient group and control group was 9.77 ± 3.37 and 9.85 ± 3.97 years, respectively. The age range studied was similar to that of Bozkurt et al. [17]. Contrary to that study, no significant difference was found between the two groups in terms of global RNFL. The similarity of total RNFL does not seem to support the hypothesis that this parameter represents a possible neuronal structure anomaly. However, thinning of the nasal and nasal superior quadrants in the left eye may indicate some specific structural differences in brain structure. The link between pathological neurodevelopment and changes in the nasal and nasal superior quadrants is not well known and further studies are needed to determine possible associations. The most striking finding for RNFL is the asymmetry between the right and left eyes. While no difference was observed in the right eye in patients with ASD, a decrease in thickness in the left eye was shown for the first time, to the best of our knowledge, in this study. There is a large literature pointing to altered brain asymmetry in ASD. Individuals with ASD have highly individualized patterns of both extreme right and left deviations in relation to the severity of symptoms, particularly in language, motor, and visuospatial regions, and research indicates that atypical lateralization may constitute a neuro-phenotype for clinically significant stratification [22]. Our results seem to support reduced leftward asymmetry. However, this result may be difficult to generalize due to the clinical heterogeneity of patients and the lack of assessment of language, motor, and visuospatial skills in patients. OCT studies to be conducted in the future, taking into account the different clinical manifestations of ASD, may be helpful in detecting the heterogeneous neuroanatomy in autism.

The second parameter investigated in our study was GCL and IPL. GCL is the part of the retina that consists of ganglion cell bodies, and IPL consists of ganglion cell dendrites. GCL and IPL in the retina may be thought of as the counterparts of cerebral grey matter because previous MRI investigations have shown considerable cerebral grey matter loss [23]. Parkinson's disease and MS patients' volumetric measurements of GCL and IPL showed declines in patient groups compared to healthy control groups [24]. Pillay et al. [25] found that GCL and IPL thickness was more sensitive clinical structural marker than RNFL in patients with early MS and that GCL correlated better with all visual parameters than RNFL thickness. Similar results were obtained in psychiatric disorders. GCL and IPL thicknesses in ASD were examined in one study. Garcia-Medina et al. [14] found that IPL was thickened in the ASD patients, while the GCL was similar in the two groups. In our study, we found decreased GCL and IPL thickness in both eyes in patients with ASD compared to controls. In addition, while the left GCL was thinner in ASD patients with intellectual disability, thinning in the left GCL and left IPL correlated with symptom severity. To the best of our knowledge, this finding is a first in this direction in the literature. According to earlier research, only 50% of the ganglion cells must be destroyed in order for an ophthalmologic examination to reveal RNFL impairment. In these studies, it was pointed out that neuronal degeneration is not reflected in the early period in RNFL and may not be a good marker [26]. Although ASD is a neurodevelopmental disorder, there are studies claiming that some patients have neurodegenerative processes. These processes are mentioned especially in cases characterized by the loss of previously acquired skills and abilities [27]. Since the past regression history is not known in the patient group, it is difficult to make a judgment about this. With this, significant thinning of GCL and IPL in ASD seems to indicate an abnormality in neuronal structure in ASD. As in other psychiatric disorders, it can be said that GCL and IPL are better biomarker candidates than RNFL, in both showing the deterioration in neuronal structure and the severity of autism symptoms. The fact that the correlation between disease severity and GCL was significant only in the left eye may be related to the altered brain asymmetry discussed above. Studies in which age-related changes can be followed longitudinally would be helpful in monitoring neuronal changes.

Dopaminergic receptors are crucial in controlling choroid development since the choroid is a highly vascularized tissue [28]. To the best of our knowledge, choroidal tissue was examined in ASD cases for the first time in this study. We found significant thinning in both eyes of patients with ASD and this result is consistent with studies showing a decrease in regional cerebral blood flow in autism [29]. According to Reynell et al. [30], autism is associated with many neurophysiological changes, and therefore it is assumed that there is an abnormal relationship between neuronal activity and brain oxygen level. Secondly, since dopamine receptors play an important role in the development of choroidal tissue, choroidal thinning may be related to the hypothesis of abnormal dopamine signaling in autism. According to these results, it can be said that there is a need for research on neurovascular coupling in ASD.

There are limitations to the present study. The first is its retrospective design, which does not allow a determination of whether these parameter changes represent a state- or a trait-marker of ASD, nor to infer information on modifications over time. Starting from the early stages, a prospective design and regular follow-up OCT measurements would give more convincing results about the nature of ASD. Second is the relatively small number of subjects enrolled and, therefore, the limited statistical power. Another limitation of our study is the lack of control measures that would increase the validity and reliability of OCT in detecting neuronal deterioration.

## Conclusions

To the best of our knowledge, this is the first study to investigate the relationship between RNFL thickness as well as GCL, IPL, and choroidal thickness with autism. Contrary to RNFL in the ASD group, IPL, GCL, and choroidal thickness were significantly reduced in both eyes. It is thought that GCL may be an important biomarker in terms of representing structural deterioration in the brain.

We think that OCT, which is a quick, simple, non-invasive, and affordable approach, can be used frequently in ASD monitoring. Additionally, these preliminary results, if confirmed, could form the basis of a possible new perspective to gain further insight into the pathophysiology of ASD.

## References

[1] American Psychiatric Association (2013). Diagnostic and statistical manual of mental disorders. https://www.psychiatry.org/psychiatrists/practice/dsm.

[2] (2023). Data & statistics on autism spectrum disorder. https://www.cdc.gov/ncbddd/autism/data.html.

[3] Li X, Zhang K, He X (2021). Structural, functional, and molecular imaging of autism spectrum disorder. Neurosci Bull.

[4] Zhao X, Zhu S, Cao Y (2022). Abnormalities of gray matter volume and its correlation with clinical symptoms in adolescents with high-functioning autism spectrum disorder. Neuropsychiatr Dis Treat.

[5] Hong SJ, Hyung B, Paquola C, Bernhardt BC (2019). The superficial white matter in autism and its role in connectivity anomalies and symptom severity. Cereb Cortex.

[6] Hadjikhani N, Joseph RM, Snyder J, Tager-Flusberg H (2006). Anatomical differences in the mirror neuron system and social cognition network in autism. Cereb Cortex.

[7] Hergüner A, Alpfidan İ, Yar A, Erdoğan E, Metin Ö, Sakarya Y, Hergüner S (2018). Retinal nerve fiber layer thickness in children with ADHD. J Atten Disord.

[8] Chua SY, Lascaratos G, Atan D (2021). Relationships between retinal layer thickness and brain volumes in the UK Biobank cohort. Eur J Neurol.

[9] Chalkias IN, Bakirtzis C, Pirounides D, Boziki MK, Grigoriadis N (2022). Optical coherence tomography and optical coherence tomography with angiography in multiple sclerosis. Healthcare (Basel).

[10] Song A, Johnson N, Ayala A, Thompson AC (2021). Optical coherence tomography in patients with Alzheimer's disease: what can it tell us?. Eye Brain.

[11] Aydin TS, Umit D, Nur OM, Fatih U, Asena K, Nefise OY, Serpil Y (2018). Optical coherence tomography findings in Parkinson's disease. Kaohsiung J Med Sci.

[12] Kalenderoglu A, Çelik M, Sevgi-Karadag A, Egilmez OB (2016). Optic coherence tomography shows inflammation and degeneration in major depressive disorder patients correlated with disease severity. J Affect Disord.

[13] Emberti Gialloreti L, Pardini M, Benassi F (2014). Reduction in retinal nerve fiber layer thickness in young adults with autism spectrum disorders. J Autism Dev Disord.

[14] García-Medina JJ, García-Piñero M, Del-Río-Vellosillo M (2017). Comparison of Foveal, macular, and peripapillary intraretinal thicknesses between autism spectrum disorder and neurotypical subjects. Invest Ophthalmol Vis Sci.

[15] Little JA, Anketell PM, Doyle L, Saunders K (2016). Investigation of retinal thickness using OCT in autism spectrum disorder. Investig Ophthalmol Vis Sci.

[16] Friedel EB, Tebartz van Elst L, Schäfer M (2022). Retinal thinning in adults with autism spectrum disorder [PREPRINT]. J Autism Dev Disord.

[17] Bozkurt A, Say GN, Sahin B (2022). Evaluation of retinal nerve fiber layer thickness in children with autism spectrum disorders. Res Autism Spectr Disord.

[18] İncekaş Gassaloğlu S, Baykara B, Avcil S, Demiral Y (2016). Validity and reliability analysis of Turkish version of childhood autism Rating Scale (Article in Turkish). Turk Psikiyatri Derg.

[19] Ünal F, Öktem F, Çetin Çuhadaroğlu F (2019). Validity of the schedule for Affective Disorders and schizophrenia for school-age children-present and Lifetime version, DSM-5 November 2016-Turkish adaptation (K-SADS-PL-DSM-5-T) (Article in Turkish). Turk Psikiyatri Derg.

[20] Jindahra P, Hedges TR, Mendoza-Santiesteban CE, Plant GT (2010). Optical coherence tomography of the retina: applications in neurology. Curr Opin Neurol.

[21] Åkerblom H, Holmström G, Eriksson U, Larsson E (2012). Retinal nerve fibre layer thickness in school-aged prematurely-born children compared to children born at term. Br J Ophthalmol.

[22] Postema MC, van Rooij D, Anagnostou E (2019). Altered structural brain asymmetry in autism spectrum disorder in a study of 54 datasets. Nat Commun.

[23] Bangalore SS, Goradia DD, Nutche J, Diwadkar VA, Prasad KM, Keshavan MS (2009). Untreated illness duration correlates with gray matter loss in first-episode psychoses. Neuroreport.

[24] Garcia-Martin E, Polo V, Larrosa JM (2014). Retinal layer segmentation in patients with multiple sclerosis using spectral domain optical coherence tomography. Ophthalmology.

[25] Pillay G, Ganger A, Singh D, Bhatia R, Sharma P, Menon V, Saxena R (2018). Retinal nerve fiber layer and ganglion cell layer changes on optical coherence tomography in early multiple sclerosis and optic neuritis cases. Indian J Ophthalmol.

[26] Quigley HA, Addicks EM (1982). Quantitative studies of retinal nerve fiber layer defects. Arch Ophthalmol.

[27] Kern JK, Geier DA, Sykes LK, Geier MR (2013). Evidence of neurodegeneration in autism spectrum disorder. Transl Neurodegener.

[28] Nickla DL, Wallman J (2010). The multifunctional choroid. Prog Retin Eye Res.

[29] Yang WH, Jing J, Xiu LJ, Cheng MH, Wang X, Bao P, Wang QX (2011). Regional cerebral blood flow in children with autism spectrum disorders: a quantitative ⁹⁹mTc-ECD brain SPECT study with statistical parametric mapping evaluation. Chin Med J (Engl).

[30] Reynell C, Harris JJ (2013). The BOLD signal and neurovascular coupling in autism. Dev Cogn Neurosci.

